# The impact of bridging education programs on internationally educated nurses becoming registered nurses in high‐income countries: A mixed‐methods systematic review

**DOI:** 10.1111/inr.13038

**Published:** 2024-08-24

**Authors:** Floro Cubelo, Anndra Parviainen, Dominika Kohanová

**Affiliations:** ^1^ School of Wellbeing and Culture, Healthcare Sector Oulu University of Applied Sciences Oulu Finland; ^2^ Department of Nursing Science University of Eastern Finland Kuopio Finland; ^3^ Department of Nursing Faculty of Social Sciences and Health Care Constantine the Philosopher University in Nitra Nitra Slovakia

**Keywords:** Bridging program, education, international nurses, migrant nurses nursing education, policy, solution

## Abstract

**Aim:**

To synthesize the current literature on the impact of bridging education programs for internationally educated nurses (IENs) from low‐ and middle‐income countries (LMICs) seeking to become registered nurses (RNs) in high‐income countries (HICs).

**Background:**

The issue of qualification pathways for IENs through bridging programs has garnered significant attention in contemporary discourse. The growing population of IENs in HICs has made it imperative to streamline the qualification process to facilitate their integration into the healthcare system.

**Methods:**

Utilizing a structured review method, we sourced data between January 2023 and April 2024 from the CINAHL, Scopus, and MEDLINE databases with no year limitations. Out of 817 studies, eight were included. The mixed‐methods systematic review was carried out by two authors who adhered to the Preferred Reporting Items for Systematic Reviews and Meta‐Analyses (PRISMA) checklist. We employed a qualitative content analysis from a feminist standpoint to assess the impact of bridging programs on the transition of IENs to become RNs.

**Results:**

Eight studies were included (mixed methods = 1, quantitative = 3, qualitative = 4). Three themes revealed important key findings. Language proficiency emerged as a critical factor influencing success in bridging programs, with IENs needing to attain a certain level of proficiency in the local language required for licensure. Enhancing nursing competence highlighted skepticism and the need for tailored educational approaches. Transitioning into the workplace emphasized cultural challenges, highlighting the importance of targeted support for continuous integration.

**Conclusion:**

Our findings revealed that despite previous higher education attainment in nursing and nursing experience in the home countries from LMICs, bridging programs aided IENs in transitioning and assimilating into the host country's healthcare employment sector.

**Implication for nursing policy and practice:**

It is essential for policymakers in the education sector to integrate language instruction, cultural sensitivity training, and adapted educational approaches into bridging programs to enhance IENs' readiness for efficient healthcare delivery.

## INTRODUCTION

The issue of qualification pathways for internationally educated nurses (IENs) via bridging programs has garnered significant attention in contemporary discourse (Aggar et al., 2020; Covell et al., 2018, 2017; Cubelo et al., 2023; Högstedt et al., 2021, 2022; Lum et al., 2016). The increasing number of IENs from low‐ and middle‐income countries (LMICs) in high‐income countries (HICs) makes it essential to streamline the qualification process for their integration into the healthcare system. These nurses play a significant role in addressing healthcare shortages in HICs, filling critical gaps in the workforce (Asamani et al., 2020, 2022; Buchan et al., 2023; Garg et al., 2022). In regions such as Asia and Africa, there is a surplus of healthcare professionals relative to available opportunities, leading to high rates of unemployment among nurses (Asamani et al., 2020, 2022; Buchan et al., 2023). Consequently, IENs often choose migration as a solution due to the challenges in securing attractive wage employment within their home countries (Buchan et al., 2023).

In recent years, several studies have highlighted the migration of IENs from LMICs to HICs, particularly to non–English‐speaking countries like Finland and Sweden (Cubelo et al., 2023; Hadziabdic et al., 2021; Högstedt et al., 2021, 2022), with some being directly recruited from abroad to address nursing shortages in these countries (Cubelo et al., 2024, 2023). This recruitment strategy raises ethical concerns, as it involves IENs from LMICs with fragile healthcare systems and existing nursing shortages. Without bilateral labor agreements, the risk of exploitation increases, as does the possibility of IENs being deskilled or unable to practice their profession in the host country (Cubelo, 2023c, 2024). However, similar strategies have been employed for years in HICs like the United Kingdom and the United States (Alexis & Shillingford, 2015; Ho, 2015).

The qualification pathway for IENs refers to the process these nurses undertake to have their education, training, and credentials recognized, validated, or aligned with the standards and requirements of the country where they intend to practice as registered nurses (RNs) or healthcare professionals (Cubelo et al., 2023; National Council of State Boards of Nursing, 2023). This includes credential evaluation by assessing transcripts and relevant materials to determine the comparability of an education program with nursing standards in the host country, covering aspects such as nursing education and entry‐to‐practice criteria (National Council of State Boards of Nursing, 2023).

Licensing and language requirements for nursing differ among HICs. In North America, the United States and Canada require candidates to pass the National Council Licensure Examination‐Registered Nurses (NCLEX‐RN) for licensure. In the Nordic region, most countries mandate a national examination, except Denmark, where the Ministry of Education and the National Board of Health share decision‐making authority. Australia, on the other hand, does not require an examination for its nursing roles (National Council of State Boards of Nursing, 2020). In the United States, evaluating IENs’ credentials by an evaluation agency or a board of nursing is imperative to verify the comparability of their education and training with specific jurisdictional standards, ensuring their safety and competence in professional practice (National Council of State Boards of Nursing, 2023).

In the context of nursing education, the bridging program is an educational initiative tailored for IENs upon their arrival in the receiving country (Cubelo, 2024). It aims to facilitate their attainment of nursing qualifications in alignment with national standards, enabling them to acquire the essential skills, knowledge, and practical experiences mandated by legislation (Cubelo et al., 2023). The duration of the bridging program depends on the regulatory prerequisites and the academic credentials of the nurse, typically lasting around 1 year (Cubelo et al., 2023; Högstedt et al., 2022).

### Aim

To synthesize the current literature on the impact of bridging education programs for IENs, seeking to become RNs in HICs. The research inquiry was explicitly defined as:

- What is the impact of bridging programs for the recognition of nursing qualifications among IENs in their country of migration?

### Methodology

This systematic review adhered to the Preferred Reporting Items for Systematic Reviews and Meta‐Analyses (PRISMA) guidelines (Page et al., 2021). It aimed to inform policymakers and decision‐makers about the implications of bridging education programs for IENs (Munn et al., 2018). A mixed‐methods systematic review using a data‐based convergent design was selected (Hong et al., 2017). The review focused on studies addressing bridging programs for the recognition of nursing qualifications among IENs in their country of migration, with the goal of guiding future research and nursing education practices. The PRISMA flow diagram was generated using the Covidence system (PRISMA, 2020).

### Literature search

**FIGURE 1:**
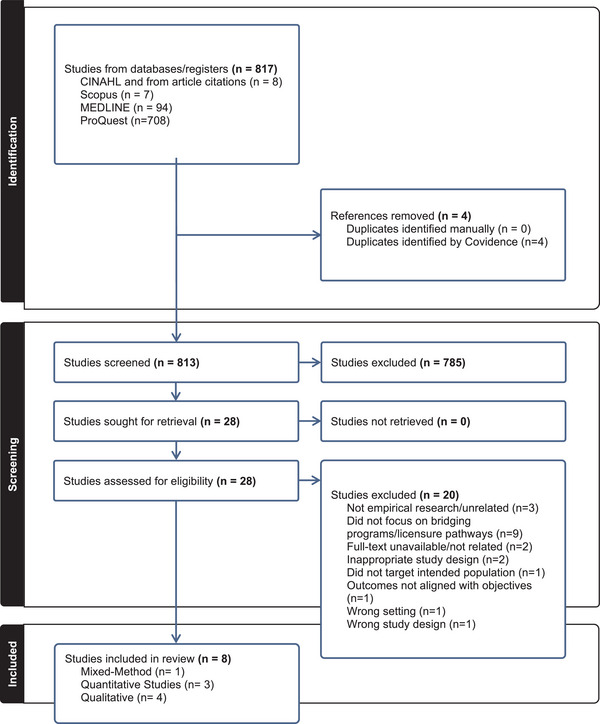
PRISMA.

The Sample, Phenomenon of Interest, Design, Evaluation, and Type of Research (SPIDER) tool was employed (see Table 1) to develop search terms aligned with the research question (Cook et al., 2012). The search was conducted across four databases: CINAHL, Scopus, ProQuest, and PubMed, using Boolean operators. Search terms included “bridg* program” or “top up educ*” and “consequence*” or “impact” or “relation*” or “associat*” AND “foreign nurs*” or “immigra* nurs*” or “international nurs*” (see Supplementary Table 1). The search was limited to English‐language publications within the last 10 years, resulting in 817 studies (see Figure 1).

**TABLE 1:** SPIDER tool terms.

Criteria	Inclusion	Exclusion
Sample	IENs seeking to become RNs	Other than IENs seeking to become RNs
Phenomenon of interest	Impact of bridging programs for the recognition of nursing qualifications among IENs in their country of migration	No reported impact of bridging programs for the recognition of nursing qualifications among IENs in their country of migration
Design	All designs	None
Research type	Empirical research	Reviews, discussion articles, study protocols, editorials, commentaries
Evaluation	Nursing perspective	Other than the nursing perspective
Time frame	Last 10 years	More than 10 years
Language	English	Languages other than English

### Data retrieval

Inclusion criteria were English‐language, peer‐reviewed articles discussing or mentioning bridging programs for obtaining an RN license. This included IENs who migrated independently to HICs without being specifically recruited for employment. The study included qualitative, quantitative, and mixed‐methods studies. Exclusion criteria were non‐English articles, literature reviews, editorial and opinion pieces, and studies not addressing the process of obtaining a nursing qualification through a bridging program.

### Screening and selection

Initially, 817 references were screened, with four duplicates removed, leaving 813 studies for title and abstract screening. Of these, 785 were excluded based on relevance to the research question. The remaining 28 studies were assessed for full‐text eligibility by two reviewers (FC and DK). After a thorough evaluation, 20 studies were excluded: three were not empirical research, nine did not focus on bridging programs or licensure pathways, two lacked full‐text availability, two had inappropriate study designs, one did not target the intended population, one had misaligned outcomes, and two were excluded due to wrong settings or study designs. Ultimately, eight studies (1 mixed‐method, 3 quantitative, and 4 qualitative) met all inclusion criteria and were included in the systematic review (see Table 2).

**TABLE 2:** Summary of studies included in the analysis.

Author (s)	Aim of the study	Methods	Country	Setting	Sample size and participants	Pathway	Relevant findings	JBI critical appraisal score
Mixed‐methods study								
Aggar et al., 2020	To explore the experiences of IENs enrolled in an authorized bridging program and examine their intentions regarding retention and pursuit of employment within the Australian healthcare sector.	Quantitative, longitudinal, mixed‐methods, and exploratory design	Australia	Regional University	9 IENs from India: 4 UK: 2 China: 1 Netherlands: 1 Philippines: 1	‐Bridging Program	The bridging program boosted confidence and understanding of the Australian healthcare system. Nursing competence, especially in clinical leadership, improved. Stress levels rose due to workload, financial strain, and social issues. All IENs intended to stay in Australia after the program. Develop an understanding of the healthcare in Australian context Improved nursing competence and clinical leadership Personal and life issues	Qualitative: 8/10 Quantitative: 6/8
Quantitative study								
Covell et al., 2017	To describe the demographic and human capital profile of IENs in Canada, examine recent changes, and determine predictors of their workforce integration	Quantitative, cross‐sectional, descriptive, correlational survey design	Canada	10 provinces and 2 territories of Canada	2280 IENs (626 joined bridging programs) **Developing country: 1287** The Philippines: 556 India: 181 China/Hong Kong: 84 Lebanon‐: 42 Jamaica: 41 Other developing countries: 383 **Developed country: 888** United Kingdom: 374 France: 131 United States: 107 Poland: 63 Australia: 44 Other developed countries: 17	‐Bridging Program	Exhibited higher language proficiency and educational qualifications, with more engagement in bridging programs and assistance services, leading to increased success in passing licensure exams and securing employment as regulated nurses. Professional experience, however, did not significantly influence job attainment, with assistance programs proving more impactful. Significantly higher numbers of participants who immigrated ≥2002 participated in bridging programs (χ^2^ = 37.85, *p* < 0.001), had help studying for the licensure exam (χ^2^ = 12.45, *p* < 0.001), and received assistance to find their first job as a regulated nurse (χ^2^ = 14.84, *p* < 0.001).	6/8
Covell et al., 2018	To evaluate IENs’ perception of bridging program benefits for nursing practice in Canada, explore variations by source country's economic status, and identify predictive human capital characteristics.	Quantitative and descriptive	Canada	Provincial regulatory bodies	359 IENs **High‐income country: 68** France: 25 Romania: 16 United Kingdom: 7 Israel: 8 Other HICs: 12 **Low‐income country: 291** The Philippines: 121 Haiti: 20 India: 22 Algeria: 17 Ivy Coast: 13 Columbia: 9 China: 9 Other low‐income countries: 80	‐Bridging Program	IENs from low‐income countries perceived bridging programs as more beneficial for enhancing cultural competency, improving language skills, and preparing for regulatory exams compared with IENs from HICs. Bridging program participation enabled IENs to acquire occupation‐specific vocabulary and gain familiarity with nursing and healthcare systems.	6/8
Högstedt et al., 2022	To compare the self‐rated professional competence, self‐efficacy, and thriving of two groups of IENs (bridging program and validation) with regular nursing students as they were about to enter the nursing profession	Quantitative, cross‐sectional, comparative design	Sweden	College and university	‐ IENs from the bridging program: 162 participants ‐ IENs from the validation process: 103 participants ‐ Regular nursing students from two higher education institutions: 312 participants	‐Bridging education program ‐Validation pathway ‐Regular admission	The study compared two groups of IENs and regular nursing students on their self‐rated professional competence, general self‐efficacy, and thriving as they were about to enter working life as RNs in Sweden. Both groups of IENs rated their competence overall as high and significantly higher than regular nursing students for all three outcome variables, with no statistically significant differences between the IEN groups.	6/8
Qualitative study								
Cubelo et al., 2023	To understand the experiences of Filipino IENs (FIENs)on their recognition and credentialing pathway in the recruitment process in Finland	Qualitative, thematic analysis	Finland	University and university hospitals	‐10 IENs from the Philippines	‐Bridging education program	‐Bridging program aided IENs in transitioning to work employment and qualified faster as RNs	8/10
Hadziabdic et al., 2021		Qualitative, descriptive design	Sweden	University	11 IENs Ethiopia: 2 Gambia: 1 Iran: 1 Belarus: 2 Syria: 2 Turkey: 1 Russia: 1 Armenia: 1		Bridging program participation did play a significant role in making the job search for IENs easier. Improve the knowledge in the Swedish healthcare system Does not see Swedish language as a barrier in life studies but recognized the importance of learning it to succeed in work life Bridging program participation did play a significant role in making the job search for IENs easier.	8/10
Högstedt et al., 2021	The aim was to examine internationally educated nurses’ experience of attending a one‐year bridging program to obtain a Swedish nursing license.	Qualitative, descriptive	Sweden	Universities	*n* = 18 IENs	Bridging education program	Studying in a new environment and language was challenging and intensive, as were adapting to a new healthcare system and relearning some nursing practices. However, attending the bridging program was also rewarding and gave feelings of happiness and pride; the nurses developed their nursing skills as well as their language and academic skills. Moreover, they became familiar with Sweden's nursing practices, healthcare system, and culture. Good support was important, but not always enough.	8/10
Lum et al., 2016	To investigate the IENs perceptions regarding the English language and nursing communication proficiency prerequisites in a Canadian bridging education program	Qualitative, grounded theory	Canada	University	‐22 IENs from the Philippines, Nigeria and from other European countries	‐Bridging education program	The bridging program in a Canadian university highlighted unexpected challenges for participants, particularly in meeting the writing demands that significantly differed from their prior nursing education.	10/10

### Data extraction

Relevant information was directly input into a spreadsheet during the data extraction process. The extracted data encompassed publication details, study objectives, methodologies, geographical focus, settings, sample sizes, participant demographics, pathways studied, and pertinent findings.

### Study risk of bias assessment

To reduce potential bias, the study implemented the following strategies: (a) all researchers actively participated in refining the study protocol; (b) two researchers conducted the literature search independently; (c) two researchers independently performed data extraction under the supervision of the senior researcher; (d) two researchers independently assessed the methodological quality of the studies; and (e) decisions at each stage were made collectively, with meetings held to decide on progressing to the next stage.

### Quality appraisal of the studies

The chosen articles underwent critical appraisal using the Joanna Briggs Institute Critical Appraisal Tools: four for qualitative studies (Lockwood et al., 2015) and four for quantitative studies (Moola et al., 2020) (see Table 2). Two independent researchers assessed the methodological quality of the studies, resolving any disagreements through discussion until consensus was achieved. Four out of five qualitative studies met almost all criteria outlined in the JBI critical appraisal tool, except for one criterion (statement locating the researcher culturally or theoretically). Only one qualitative study (Lum et al., 2016) met all criteria. None of the quantitative studies met all the criteria as reported in the JBI critical appraisal tool; these studies lacked the assessment of confounding factors (see Supplementary Table 2).

### Data analysis and synthesis

Using the convergent integrated approach, we combined qualitative and quantitative data, including mixed‐method research (Stern et al., 2020). In this systematic literature review, we employed Mayring's (2014) qualitative content analysis (QCA) method to categorize and interpret textual data, identifying prevalent patterns, themes, and underlying meanings. The QCA was suitable as it allowed for the integration of both quantitative and qualitative data (Vaismoradi et al., 2013).

The QCA process involved several systematic steps: initially coding the text to identify themes and assign relevant excerpts, defining these themes to select pertinent material, revising categories and themes in comparison with research questions, conducting final coding to refine and develop main themes, and presenting the results in a summative and narrative manner, providing a comprehensive overview of the findings (Mayring, 2014). For the quantitative data, we performed qualitization, converting it into narrative interpretations (Stern et al., 2020) to reduce potential errors from assigning numerical values to qualitative data, ensuring a more accurate representation of the findings (JBI, 2014).

Additionally, we adopted a feminist standpoint to discuss the implications of bridging programs for IENs, acknowledging social issues and power dynamics. This approach emphasized equality and social justice, recognizing the influence of gender, history, and politics on knowledge (Aranda, 2018), and highlighting the impact of social hierarchies and oppression (Brown et al., 2013). This perspective integrates academic and political views to advocate for a fairer society (Wilson, 2023).

## RESULTS

The selected articles included 2967 IENs, offering extensive geographical diversity, excluding local regular students who were part of the study. Participating countries with established bridging programs included Sweden (2), Canada (3), Finland (1), and Australia (1). The majority of IENs, with clearly stated demographics from the selected studies, originated from the Philippines, India, and the United Kingdom. The results highlighted significant differences in host countries' expectations for nursing licensure, particularly regarding practice and professional language competencies. These findings underscored the need for bridging programs to address the substantial gaps between past and future practice competencies for IENs.

In Australia, findings revealed that participation in the bridging program significantly increased confidence and understanding of the Australian healthcare system among IENs, with improvements noted in nursing competence, particularly in clinical leadership (Aggar et al., 2020). Similarly, in Canada, IENs with higher language proficiency and educational qualifications, as well as those engaging in bridging programs and assistance services, exhibited increased success in passing licensure exams and securing employment as regulated nurses, although professional experience did not significantly influence job attainment (Covell et al., 2018, 2017).

Findings from Finland indicated that bridging programs aided IENs in transitioning to work and qualifying faster as RNs (Cubelo et al., 2023). Additionally, challenges were highlighted in a Canadian bridging education program, particularly in meeting the writing demands, which significantly differed from participants' prior nursing education (Lum et al., 2016). While in Sweden, both groups of IENs rated their overall competence as high and significantly higher than regular nursing students for all three outcome variables, with no statistically significant differences between the IEN groups (Högstedt et al., 2022). They also had a positive experience in the overall implementation of the bridging program (Hadziabdic et al., 2021).

### Theme 1: Language requirement as a bridge and gap

The IENs originating from LMICs perceive participation in bridging programs as beneficial for enhancing their proficiency in the official language of the host country compared with those from high‐income nations (Covell et al., 2018). Although IENs found language to be a challenge in attending a bridging program in a non–English‐speaking country (Cubelo et al., 2023; Högstedt et al., 2021), they do not view the Swedish language as a hindrance in academic pursuits. Instead, they recognize its significance for achieving success in professional endeavors, such as learning occupation‐related vocabulary (Covell et al., 2018). Covell et al. (2017) and Hadziabdic et al. (2021) suggested that the educational background and language skills of IENs likely influence their performance on licensure exams, with many migrants possessing a proficient level of English, thus gaining an advantage.

A discernible contrast in this study revealed that language prerequisites differ between bridging programs offered in countries where English is not the primary language and those where it is the predominant mode of communication, presenting notable obstacles for IENs (Cubelo et al., 2023; Hadziabdic et al., 2021; Högstedt et al., 2021, 2022; Lum et al., 2016). In this context, when IENs opt for a bridging program, they must provide evidence of their Swedish language proficiency during the application process (Hadziabdic et al., 2021; Högstedt et al., 2022). In contrast, those who pursue the validation pathway have the flexibility to learn the language at any stage but must certify their competency before applying for the nursing license (Högstedt et al., 2022), potentially impeding their ability to successfully complete the program and obtain recognition for their nursing credentials (Cubelo et al., 2023; Högstedt et al., 2022). To improve effective communication, incorporating acronyms and terminology to optimize communication efficacy and foster precise information transmission among healthcare practitioners was crucial for IENs to learn (Lum et al., 2016).

### Theme 2: Enhancing nursing competence

During the bridging program, IENs perceive it as instrumental in acquiring technical knowledge, familiarizing themselves with work routines as RNs, and gaining insights into the healthcare system of the host country (Aggar et al., 2020; Cubelo et al., 2023; Hadziabdic et al., 2021; Högstedt et al., 2021). In a comprehensive Canadian study by Covell et al. (2017), it was found that professional experience and study assistance significantly predicted the success of IENs in passing the licensure exam on their first attempt, even for those who did not attend the bridging program. Additionally, the bridging programs positively impacted IENs by enhancing their cultural competency, expanding their knowledge about the nursing profession, and improving their professional skills within the local healthcare setting (Covell et al., 2018).

The IENs employed in Canada expressed doubts regarding the necessity of reassessing their nursing competence and pursuing further education, questioning the justification behind these requirements (Lum et al., 2016). Furthermore, data indicated that IENs in Sweden reported lower proficiency in value‐based nursing care, which encompasses ethical principles, patient‐focused care, and collaborative work (Högstedt et al., 2022). They perceived themselves as relatively less competent in aspects related to ethics, patient‐centered care, and efficient teamwork within this domain (Högstedt et al., 2022), suggesting that these elements should be incorporated into the bridging program (Hadziabdic et al., 2021). Additionally, there is a recognized need for IENs to enhance their therapeutic communication skills, which are critical for delivering patient‐centered care (Lum et al., 2016). Conversely, some IENs felt that certain courses within the bridging program, such as those related to medical subjects and pharmacology, seemed redundant as they overlapped with content previously covered in their nursing education or professional experience in their countries of origin (Hadziabdic et al., 2021).

In Finland, the bridging program was coordinated between the employer and higher education institution when IENs were recruited from abroad, highlighting some differences in nursing practices between the source and host countries (Cubelo et al., 2023). Importantly, the IENs were aware of these differences before undertaking the program, but they experienced conflicts in terms of their values and skills as they worked toward obtaining their qualification (Cubelo et al., 2023; Lum et al., 2016).

Similarly, in Sweden, IENs had a positive experience attending the bridging program, as it provided them with the opportunity to utilize other IENs’ skills and competencies to advance in the program. They planned to pursue becoming specialist nurses after gaining years of clinical experience (Hadziabdic et al., 2021). The bridging program effectively upgraded their professional competencies to meet the country's standards (Covell et al., 2017; Högstedt et al., 2021, 2022).

### Theme 3: Bridge to work–life transition

The bridging program was beneficial for IENs in understanding employment conditions, including salaries, work schedules, and benefits (Covell et al., 2018). Many expressed a desire to stay and practice in the host country after completing the program (Aggar et al., 2020). Overall, it served as a pathway for obtaining licensure as an RN (Covell et al., 2018, 2017; Hadziabdic et al., 2021; Högstedt et al., 2021, 2022). However, even after becoming regulated nurses, challenges in securing employment were encountered, though bridging program participants faced fewer difficulties (Covell et al., 2018).

Experienced nurses mandated to undertake additional bridging education faced significant challenges, as cultural dissonance could impede their advancement in bridging programs and job opportunities (Cubelo et al., 2023; Lum et al., 2016). Participation in bridging programs and assistance from social networks were significant predictors of employment difficulty for IENs in Canada (Covell et al., 2017).

Högstedt and colleagues (2022 et al. (2022) noted increased levels of self‐efficacy and thriving among nurses enrolled in bridging programs, potentially augmenting their competencies and general welfare in the occupational context (Cubelo et al., 2023; Högstedt et al., 2022). Similarly, a bridging program facilitated IENs in obtaining nursing assistant positions and gaining work experience as nurses in Sweden (Högstedt et al., 2021). However, Lum and colleagues (2016)) reported a lack of understanding among IENs regarding the relevance of acquiring new skills and the necessity of additional education for future nursing practice. Nonetheless, most participants had prior exposure to the work culture, having been employed as nursing assistants and practical nurses.

## DISCUSSION

The current study employed a feminist framework to gain a comprehensive understanding of the implications of bridging programs on the qualification attainment of IENs as RNs. Within this context, the requirement of language proficiency for admission into a bridging program in a nonnative English‐speaking country was not deemed essential for demonstrating the competencies of IENs, as it could be developed during the educational program. Considering that four of the selected studies took place in Finland and Sweden (Cubelo et al., 2023; Hadziabdic et al., 2021; Högstedt et al., 2021, 2022), it was crucial to recognize that nurses educated outside the region primarily encountered the challenge of passing the language proficiency test. They also faced additional obstacles in having their nursing qualifications recognized in the destination country, despite possessing sufficient years of nursing experience. Even in English‐speaking countries, insufficient language proficiency posed challenges for IENs, hindering their ability to deliver comprehensive care (Lum et al., 2016).

In the context of feminism, the unfavorable implications concerning the educational backgrounds of IENs educated in LMICs signify a form of social injustice and pose a potential risk of inequalities such as deskilling (Cubelo et al., 2023; Salami et al., 2018; Tayaben & Younas, 2020).

Numerous studies have consistently emphasized the significant role of language barriers as a prominent source of frustration and disappointment among IENs. Within this context, various issues arise, including instances of colleagues employing slang to undermine IENs and experiencing discrimination due to their noticeable accents (Brunton & Cook, 2018; Buttigieg et al., 2018; Cubelo, 2023b; Iheduru‐Anderson & Wahi, 2018). These findings underscore the detrimental implications of language‐related challenges on collegiality, patient safety, and professional development.

Considering the implications of a bridging program, it is important to prioritize the development of language and communication skills for IENs, who often use English as a second language and possess significant experience and expertise. By focusing on improving these skills, bridging programs can contribute to better patient outcomes, promote collegiality among healthcare professionals, and facilitate the advancement of IENs in their career pathways (Cubelo, 2024; Cubelo et al., 2023; Hadziabdic et al., 2021; Högstedt et al., 2021, 2022). In nonnative English‐speaking countries, incorporating language teachers within clinical skills labs, high‐fidelity simulations, and training supervision can effectively address language‐related aspects without compromising the core nursing content (Cubelo, 2023a).

One best practice in the United States is the use of credentialing bodies to help IENs systematically get recognized as RNs before moving to the country. Typically, IENs must submit their credentials to a recognized body like the Commission on Graduates of Foreign Nursing Schools (CGFNS) to ensure their qualifications are equivalent to those of U.S.‐educated nurses. This credentialing process is a prerequisite before IENs can take the NCLEX‐RN, the national licensing exam for RNs. Therefore, IENs must possess an RN qualification before being licensed to practice in the United States. Additionally, agencies commonly recruit IENs to help them navigate this process, including obtaining credentials and preparing for the NCLEX‐RN (CGFNS International, 2019). Although this is a good practice, it can be challenging in non—English‐speaking countries where learning the local language, such as Finnish and Swedish, can be difficult (Cubelo et al., 2023; Högstedt et al., 2022).

Within the context of a bridging program, IENs have demonstrated their proficiency and confidence in professional competencies (Aggar et al., 2020; Cubelo et al., 2023; Hadziabdic et al., 2021; Högstedt et al., 2021, 2022). However, there is a need for additional guidance and education, particularly in the areas of value‐based nursing, patient‐centered care, and teamwork (Högstedt et al., 2022). These skills are crucial for successful integration and practice in Western countries (Högstedt et al., 2022). It is worth considering that IENs coming from LMICs may have previous nursing experience in environments characterized by a high degree of hierarchy. Consequently, addressing these cultural differences and providing targeted education can support their transition and ensure the development of the necessary skills for their nursing practice in the new context.

The selected studies revealed that migration decisions were often influenced by women's caregiving roles within the family, an important observation from a feminist standpoint. A considerable number of participants migrated for family reasons (Högstedt et al., 2022). Additionally, a significant proportion of the participants had obtained undergraduate or graduate degrees in their countries of origin (Cubelo et al., 2023; Högstedt et al., 2022; Lum et al., 2016), highlighting their academic accomplishments and vocational credentials. The nursing expertise that IENs brought from their countries of origin demonstrated their proficiency and significant contributions to the healthcare industry (Cubelo et al., 2023; Högstedt et al., 2022; Lum et al., 2016). The results illustrated the intricate interplay between gender, familial dynamics, educational background, and occupational experiences in the context of global migration for IENs.

Moreover, the bridging programs studied were designed to prepare IENs to become RNs in the host country, effectively providing them with essential competencies. The efficacy of these programs in enhancing the confidence and self‐perceived proficiency of IENs (Högstedt et al., 2022) highlighted their importance in facilitating the transition and integration of IENs into the healthcare system by addressing their challenges (Cubelo et al., 2023; Hadziabdic et al., 2021; Högstedt et al., 2022).

The study examined not only the experiences of IENs but also the challenges faced by individuals enrolled in bridging programs in both non‐English and English‐speaking countries. These individuals encountered unforeseen challenges in meeting the academic language requirements of the program, which were markedly different from their previous nursing education (Cubelo et al., 2023; Hadziabdic et al., 2021; Högstedt et al., 2022; Lum et al., 2016). The findings highlighted the importance of addressing the unique barriers and needs of IENs, particularly in language and communication skills. Providing targeted support and resources was essential to help them effectively adapt to academic and professional demands.

Additionally, the primary outcome of this research emphasized the significant impact of culture shock on the integration of IENs into the work environment. Acknowledging and mitigating the potential obstacles and difficulties related to cultural adjustment was imperative in facilitating the smooth integration and occupational attainment of IENs (Aggar et al., 2020; Cubelo et al., 2023; Lum et al., 2016).

### Study limitations and recommendations

Most of the studies included in the mixed‐methods review that met the criteria are qualitative. Additionally, limiting the selected articles to those published in English might exclude significant scientific contributions in other languages, such as Finnish and Swedish. Furthermore, the availability of scientific articles within the academic databases of the researchers' institution might have resulted in the omission of other important literature.

Further research must explore the impact of bridging programs in non–English‐speaking countries by understanding the perspectives and experiences of IENs who attended these programs. This research should focus on how bridging programs should be structured and whether a national model under government oversight will ease the transition phase for IENs, particularly those with years of experience in the clinical field. It is also essential to determine whether it is necessary to arrange these programs for IENs with extensive clinical experience.

### Implications for nursing policy and practice

There is a necessity to tailor bridging programs to address the specific language and cultural challenges faced by IENs, ensuring effective integration and enhancing their competency in delivering patient‐centered care in diverse healthcare settings. Nonnative English‐speaking countries recruiting IENs from LMICs need to provide adequate learning and testing facilities for the language officially used by the host countries.

Including nurse educators in the recruitment process can help assess the skills and competencies of IENs using the national standards of the country of migration. Additionally, nurse managers should create clear career pathways that enable the complete utilization of the previous knowledge and skills of recruited IENs.

## CONCLUSION

The study's findings indicated that IENs face difficulties in English‐speaking countries, even though English served as the language of instruction. These challenges were also present in non–English‐speaking countries and bridging programs that mandate a certain level of language proficiency for admission. The language requirement was not only an aptitude requirement but also a capacity test for therapeutic communication, which is crucial for delivering patient‐centered care. Although IENs possess prior nursing experience, there is still a need for enhancing holistic and patient‐centered care.

Nurse educators should familiarize themselves with the nursing curriculum and healthcare systems of LMICs when assigning clinical practicums for IENs. Cultural sensitivity is essential to provide appropriate guidance to IENs. Insufficient knowledge could impede the effective guidance and smooth integration of IENs into the nursing field.

In this context, it is essential to adopt a feminist approach when addressing the challenges encountered by IENs, as most are coming from LMICs. To support the growth and success of IENs, it is recommended to prioritize language skill development, involve language teachers in clinical skills laboratories, and consider international nursing experiences when assigning clinical practicums. This approach can foster an inclusive environment, enhancing both individual nurses' well‐being and the provision of culturally sensitive and patient‐centered care.

## ETHICAL CONSIDERATION

Ethics permit was not necessary for this study.

## AUTHOR CONTRIBUTIONS

Study design: FC, AP, DK; data collection: FC, DK; data analysis: FC, AP, DK; manuscript writing: FC, AP, DK; critical revisions for important intellectual content: FC, AP, DK.

## CONFLICT OF INTEREST STATEMENT

The authors declare no conflict of interest.

## Supporting information

Supporting Information

Supporting Information
