# Supplementary material for: The impact of bridging education programs on internationally educated nurses becoming registered nurses in high‐income countries: A mixed‐methods systematic review
Source: Int Nurs Rev. 2024 Aug 24;72(2):e13038. doi: 10.1111/inr.13038 (PMC11969313; doi:10.1111/inr.13038)
Supplement: Supplementary file 1 — Supporting Information [file INR-72-0-s001.docx]

| PubMed | English language  peer-reviewed  10 years old | bridg* program or top up educ* and consequence* or impact or relation* or associat* AND foreign nurs* or immigra* nurs* or international nurs* | 94 |
| --- | --- | --- | --- |
| Scopus | English language  peer-reviewed  10 years old | bridg* program or top up educ* and consequence* or impact or relation* or associat* AND foreign nurs* or immigra* nurs* or international nurs* | 7 |
| CINAHL and from articles citation | English language  peer-reviewed  10 years old | bridg* program or top up educ* and consequence* or impact or relation* or associat* AND foreign nurs* or immigra* nurs* or international nurs* | 8 |
| ProQuest | English language  peer-reviewed  10 years old | bridging program AND internationally educated nurse OR migrant nurse OR foreign nurse | 708 |

Supplementary Table 1. Search results from academic databases.
