# Supplementary material for: The impact of bridging education programs on internationally educated nurses becoming registered nurses in high‐income countries: A mixed‐methods systematic review
Source: Int Nurs Rev. 2024 Aug 24;72(2):e13038. doi: 10.1111/inr.13038 (PMC11969313; doi:10.1111/inr.13038)
Supplement: Supplementary file 2 — Supporting Information [file INR-72-0-s002.docx]

**Supplementary Table 2. Quality Appraisal.**

**Quality appraisal of qualitative studies**

| **Criteria** | **Lum et al. (2016)** | **Cubelo et al. (2023)** | **Hadziabdic et al. (2021)** | **Högstedt et al. (2021)** | **Aggar et al. (2020)** |
| --- | --- | --- | --- | --- | --- |
| Is there congruity between the stated philosophical perspective and the research methodology? | Y | Unclear | Unclear | Unclear | Y |
| Is there congruity between the research methodology and the research question or objectives? | Y | Y | Y | Y | Y |
| Is there congruity between the research methodology and the methods used to collect data? | Y | Y | Y | Y | Y |
| Is there congruity between the research methodology and the representation and analysis of data? | Y | Y | Y | Y | Y |
| Is there congruity between the research methodology and the interpretation of results? | Y | Y | Y | Y | Y |
| Is there a statement locating the researcher culturally or theoretically? | Y | Unclear | Unclear | Unclear | N |
| Is the influence of the researcher on the research, and vice- versa, addressed? | Y | Y | Y | Y | N |
| Are participants, and their voices, adequately represented? | Y | Y | Y | Y | Y |
| Is the research ethical according to current criteria or, for recent studies, and is there evidence of ethical approval by an appropriate body? | Y | Y | Y | Y | Y |
| Do the conclusions drawn in the research report flow from the analysis, or interpretation, of the data? | Y | Y | Y | Y | Y |

**Quality appraisal of analytical cross-sectional studies**

| **Criteria** | **Högstedt et al. (2022)** | **Covell et al. (2018)** | **Covell et al. (2017)** | **Aggar et al. (2020)** |
| --- | --- | --- | --- | --- |
| Were the criteria for inclusion in the sample clearly defined? | Y | Y | Y | Y |
| Were the study subjects and the setting described in detail? | Y | Y | Y | Y |
| Was the exposure measured in a valid and reliable way? | Y | Y | Y | Y |
| Were objective, standard criteria used for measurement of the condition? | Y | Y | Y | Y |
| Were confounding factors identified? | N | N | N | N |
| Were strategies to deal with confounding factors stated? | N | N | N | N |
| Were the outcomes measured in a valid and reliable way? | Y | Y | Y | Y |
| Was appropriate statistical analysis used? | Y | Y | Y | Y |
